# Can Radiomics Provide Additional Information in [^18^F]FET-Negative Gliomas?

**DOI:** 10.3390/cancers14194860

**Published:** 2022-10-05

**Authors:** Katharina von Rohr, Marcus Unterrainer, Adrien Holzgreve, Maximilian A. Kirchner, Zhicong Li, Lena M. Unterrainer, Bogdana Suchorska, Matthias Brendel, Joerg-Christian Tonn, Peter Bartenstein, Sibylle Ziegler, Nathalie L. Albert, Lena Kaiser

**Affiliations:** 1 Department of Nuclear Medicine, University Hospital, LMU Munich, 81377 Munich, Germany; 2 Department of Radiology, University Hospital, LMU Munich, 81377 Munich, Germany; 3 Department of Neurosurgery, Sana Hospital, 47055 Duisburg, Germany; 4 German Center for Neurodegenerative Diseases (DZNE), 81377 Munich, Germany; 5 Munich Cluster for Systems Neurology (SyNergy), 81377 Munich, Germany; 6 Department of Neurosurgery, University Hospital, LMU Munich, 81377 Munich, Germany; 7 German Cancer Consortium (DKTK), Partner Site Munich, German Cancer Research Center (DKFZ), 69120 Heidelberg, Germany; 8 Bavarian Cancer Research Center (BZKF), 91054 Erlangen, Germany

**Keywords:** amino acid PET, FET PET, glioma, FET negative, photopenic, radiomics

## Abstract

**Simple Summary:**

Amino acid positron emission tomography (PET) complements standard magnetic resonance imaging (MRI) since it directly visualizes the increased amino acid transport into tumor cells. Amino acid PET using O-(2-[^18^F]fluoroethyl)-L-tyrosine ([^18^F]FET) has proven to be relevant, for example, for glioma classification, identification of tumor progression or recurrence, or for the delineation of tumor extent. Nevertheless, a relevant proportion of low-grade gliomas (30%) and few high-grade gliomas (5%) were found to show no or even decreased amino acid uptake by conventional visual analysis of PET images. Advanced image analysis with the extraction of radiomic features is known to provide more detailed information on tumor characteristics than conventional analyses. Hence, this study aimed to investigate whether radiomic features derived from dynamic [^18^F]FET PET data differ between [^18^F]FET-negative glioma and healthy background and thus provide information that cannot be extracted by visual read.

**Abstract:**

The purpose of this study was to evaluate the possibility of extracting relevant information from radiomic features even in apparently [^18^F]FET-negative gliomas. A total of 46 patients with a newly diagnosed, histologically verified glioma that was visually classified as [^18^F]FET-negative were included. Tumor volumes were defined using routine T2/FLAIR MRI data and applied to extract information from dynamic [^18^F]FET PET data, i.e., early and late tumor-to-background (TBR_5–15_, TBR_20–40_) and time-to-peak (TTP) images. Radiomic features of healthy background were calculated from the tumor volume of interest mirrored in the contralateral hemisphere. The ability to distinguish tumors from healthy tissue was assessed using the Wilcoxon test and logistic regression. A total of 5, 15, and 69% of features derived from TBR_20–40_, TBR_5–15_, and TTP images, respectively, were significantly different. A high number of significantly different TTP features was even found in isometabolic gliomas (after exclusion of photopenic gliomas) with visually normal [^18^F]FET uptake in static images. However, the differences did not reach satisfactory predictability for machine-learning-based identification of tumor tissue. In conclusion, radiomic features derived from dynamic [^18^F]FET PET data may extract additional information even in [^18^F]FET-negative gliomas, which should be investigated in larger cohorts and correlated with histological and outcome features in future studies.

## 1. Introduction

In neurooncology, magnetic resonance imaging (MRI) represents the gold standard when diagnosing gliomas, monitoring treatment, and assessing treatment response. Due to its widespread use, high spatial resolution, and good contrast in soft tissue, MRI is the method of choice. However, the use of MRI has limitations. First, the differentiation between glioma entities, as well as between neoplastic and other lesions, particularly after treatment, causes difficulties. Chemo- and radiotherapy can induce post-therapeutic effects, including radiation necrosis and edema, which are difficult to differentiate from tumor progression or recurrence [1]. Additionally, MRI can be unreliable when determining tumor size or growth. Recent studies have shown that when comparing the tumor volume assessed in positron emission tomography (PET) and MRI, substantial spatial differences can be found [2]. Therefore, according to the RANO working group, the additional use of radiolabeled amino acids is recommended [3]. O-(2-[^18^F]fluoroethyl)-L-tyrosine ([^18^F]FET) PET can be used to detect biologically active tumor parts, and the low uptake in normal brain parenchyma leads to an enhanced tumor-to-brain contrast, thus enabling an accurate differentiation of tumor from healthy tissue. Previous studies have shown that amino acid PET can identify the glioma extent more reliably than MRI [4]. However, around 30% of low-grade gliomas and 5% of high-grade gliomas show indifferent or even decreased amino acid uptake compared to healthy tissue [5]. The pathomechanism for this phenomenon is still unclear. Although previous studies suggest that the L-amino acid transporter 1 (LAT1) primarily promotes [^18^F]FET uptake, [^18^F]FET-negative gliomas did not show a reduced LAT1 expression in immunohistochemistry [6,7]. It remains indistinct whether [^18^F]FET-negative gliomas have a favorable prognosis or not. One study reported that a small number of patients with photopenic gliomas, meaning gliomas with lower amino acid uptake than healthy tissue, even have an inferior prognosis compared to isometabolic gliomas [8]. However, these findings need to be further validated in larger patient cohorts also with respect to the underlying biological mechanisms.

Radiomics, as a subdiscipline of artificial intelligence, is based on the extraction of quantitative features from medical images such as MRI, PET, or computed tomography (CT). Radiomics is increasingly used to noninvasively determine lesion properties such as the degree of tumor heterogeneity or shape, providing additional information from routinely acquired images [9,10,11]. By combining pathomolecular parameters and radiomic features, prognostic models can be generated, enabling automation of various steps within the diagnostic routine. Radiomics is of increasing interest, as it often achieves higher diagnostic accuracy than conventional PET image parameters alone [12,13].

The aim was to evaluate whether [^18^F]FET-negative gliomas contain information that cannot be extracted by conventional visual read but by radiomic feature analysis and to what extent radiomics from conventional late static, early static, and dynamic time-to-peak PET images may help to identify tumor tissue.

## 2. Materials and Methods

### 2.1. Patients

For this retrospective study, we included 46 patients with a newly diagnosed, histologically verified glioma who had undergone a dynamic [^18^F]FET PET scan at the Department of Nuclear Medicine of the University Hospital, LMU Munich, and showed tumoral [^18^F]FET uptake equal or below the background activity. Gliomas were visually classified as [^18^F]FET^-^negative by trained nuclear medicine physicians. All patients signed written informed consent as part of the clinical routine, and the local ethics committee approved the retrospective analysis of the data (approval number 604-16).

Tissue samples were obtained from stereotactic biopsy or surgery and used for glioma classification according to the 2021 WHO guidelines [14]. Molecular markers such as isocitrate dehydrogenase (IDH) mutation status and codeletion of chromosome arms 1p and 19q (1p/19q codeletion) were obtained in accordance with previous studies [15,16]. Furthermore, the O^6^-methylguanine-DNA-methyltransferase (MGMT) promoter methylation was determined [16].

### 2.2. [^18^F]FET PET Imaging

Dynamic [^18^F]FET PET scans were acquired with an ECAT EXACT HR+ scanner (Siemens Healthineers, Erlangen, Germany) after intravenous bolus injection of a standard dose of 185 MBq of [^18^F]FET, according to standard protocols [17]. Dynamic emission data were recorded 0–40 min post-injection (p. i.) in 3D mode with 16 frames (7 × 10 s, 3 × 30 s, 1 × 2 min, 3 × 5 min, and 2 × 10 min). Image reconstruction and processing, including motion correction, were performed as described previously [18].

### 2.3. MR Imaging

All patients underwent routine MRI prior to tissue sampling with a 1.5 T or 3.0 T magnet before and after the injection of a gadolinium-based contrast agent (MultiHance, Bracco Imaging, Milan, Italy). Axial T1- and T2-weighted and FLAIR sequenced were acquired.

### 2.4. Delineation of Tumor and Background Volumes

All volumes of interest (VOIs) were defined using PMOD View tool (version 3.5, PMOD Technologies LLC, Zurich, Switzerland). First, PMOD Fusion tool (version 3.5) was used to coregister and resample each patient’s T2/FLAIR image to the corresponding [^18^F]FET PET image. Since for [^18^F]FET-negative gliomas the VOI cannot be defined within PET images, manual contouring of signal hyperintensity was performed in the T2/FLAIR weighted images. This VOI was then applied to extract information from dynamic [^18^F]FET PET data.

A crescent-shaped background VOI manually drawn in the contralateral hemisphere served as reference tissue for the quantification of tumor-to-background ratios (TBR). This procedure has proven to yield most stable background values for quantification purposes with the lowest inter- and intra-reader variability [19].

**Figure 1:**
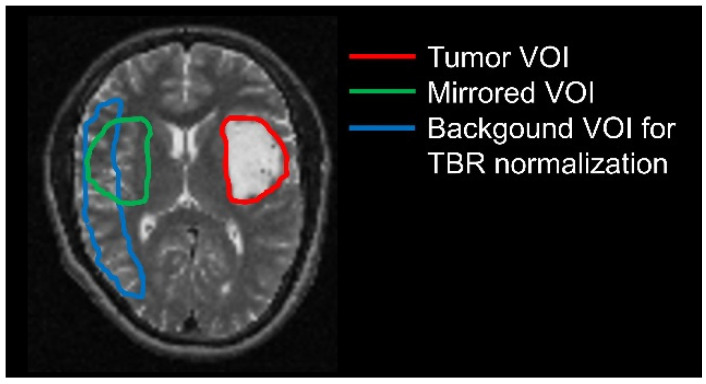
Volumes-of-interest used for analyses of [^18^]FET-negative gliomas: tumor VOI defined manually within T2/FLAIR images (red); tumor VOI mirrored to the contralateral site (green); background VOI for image normalization (blue).

A second background VOI was obtained by mirroring the manually generated tumor VOI to the contralateral unaffected brain tissue, excluding the ventricle (Figure 1). This second VOI was chosen as it enables a direct comparison between healthy and tumor tissue for radiomics analyses.

### 2.5. Generation of Parametric Images

Static early 5–15 min p. i. and standard 20–40 min p. i. summation images were calculated from dynamic PET data and normalized with the respective mean uptake within the crescent-shaped background VOI, yielding early TBR_5–15_ and standard TBR_20–40_ images. In addition to quantification using static images, parametric images containing information on the peak time point (time-to-peak, TTP) of each voxel’s time-activity curve were created as described previously [12,18].

### 2.6. Extraction of Radiomic Features

Different kinds of quantitative features were extracted from medical images. These were calculated directly from image intensities or intensity histograms (first order), from a second image obtained by the application of image filters aiming to describe image texture, or from the tumor label mask yielding shape information on the segmented tumor VOI. These radiomic features were extracted with the open-source Python (version 3.8) package pyradiomics (version 3.0.1 [18]). The default first order (n = 18) and texture (n = 75) features were included. Most of the feature definitions implemented in pyradiomics comply with definitions by the Image Biomarker Standardization Initiative (IBSI) [20].

### 2.7. Statistical Analyses

Results are provided as mean and standard deviation and/or median and range. Statistical analysis was performed with IBM SPSS Statistics (version 28). For each radiomic feature, differences between tumor and healthy tissue were evaluated using Wilcoxon test for paired non-parametric variables. *p*-values below 0.05 were considered statistically significant.

Analyses were performed for the entire patient cohort of [^18^F]FET-negative gliomas and for the subgroup of only isometabolic gliomas after exclusion of cases with visually photopenic defects.

### 2.8. Differentiation of Tumor from Healthy Tissue Using Logistic Regression

In addition to the direct comparison of single radiomic features in [^18^F]FET-negative gliomas and healthy tissue, the ability to differentiate tumors from healthy tissue using machine learning was addressed. Tumor was distinguished from healthy tissue for the whole cohort and for isometabolic and photopenic gliomas separately. The respective classification procedure was implemented using python (version 3.8) and scikit-learn package (version 1.0.2).

Machine learning was performed using logistic regression (LR) classifiers optimizing the area under the receiver operating characteristic curve (AUC). LR was applied in balanced mode, which allows for automatically adjusting sample weights according to class frequencies and thus reducing the effect of imbalanced input data. Liblinear solver was applied with L2 regularization, thus allowing for preventing overfitting and handling multicollinearity. The remaining settings of the LR classifiers were set to the default values defined in scikit-learn.

The machine learning pipeline included the following steps: (1) standardization of features by removing the mean and scaling to unit variance, (2) exclusion of features with zero variance, (3) tuning of the inverse regularization strength C, which adjusts L2 penalty using cross-validation (CV). In order to report cross-validated scores, this pipeline was inserted into an outer CV loop of a nested-CV procedure. Inner and outer CV loops were chosen to have 50 repeats and 5 folds with stratified splits containing equal distributions of the class labels in each fold. The high number of random splits in repeated CV improves the robustness of performance estimates.

## 3. Results

### 3.1. Patient Characteristics

A total of 46 patients (median age 35 years, range 16–72 years) were enrolled in this study. A total of 29 gliomas were classified as astrocytoma, *IDH* mutant (22 WHO grade two, 6 WHO grade three, 1 WHO grade four), 5 gliomas as an oligodendroglioma, *IDH* mutant with 1p/19q codeletion (5 WHO grade two), 11 gliomas as glioblastoma, *IDH* wildtype, and 1 glioma as WHO grade one. In three cases, the *IDH* mutation status could not be determined due to a lack of tumor tissue for reevaluation (two cases histologically classified as diffuse astrocytoma WHO grade two, one case as anaplastic astrocytoma WHO grade three according to WHO 2016 classification). A total of 17 patients presented with photopenic defects and 29 with isometabolic gliomas, which are not visually identifiable in [^18^F]FET PET images.

### 3.2. Differences between [^18^F]FET-Negative Tumor and Healthy Tissue—Overview

The numbers of significant features (*p* < 0.05) according to the Wilcoxon test for paired non-parametric variables are given in Table 1. Notably, the largest proportion of significant features comprised parameters derived from TTP images (> 60%) and was lower in static early TBR_5–15_ and standard TBR_20–40_ images, particularly in the subgroup of isometabolic gliomas. All results from the Wilcoxon test are provided in Supplementary Table S1.

### 3.3. Differences between Isometabolic Tumor and Healthy Tissue

In standard TBR_20–40_ images, 8 out of 11 significant features were first-order features quantifying the magnitude of voxel values, which was higher in tumors compared to healthy tissue. Among texture features, for example, the size-zone non-uniformity, a feature measuring the variability of size zone volumes, presented with significantly lower mean values in healthy tissue, indicating that tumor tissue, although visually [^18^F]FET-negative, shows more heterogeneity.

In early TBR_5–15_ images, only three features presented with a *p*-value < 0.05: skewness, cluster shade, and small area high gray level emphasis. For all features, the tumor showed higher mean values, indicating, e.g., a higher asymmetry of the intensity distribution or the gray level co-occurrence matrix in the tumor.

**Figure 2:**
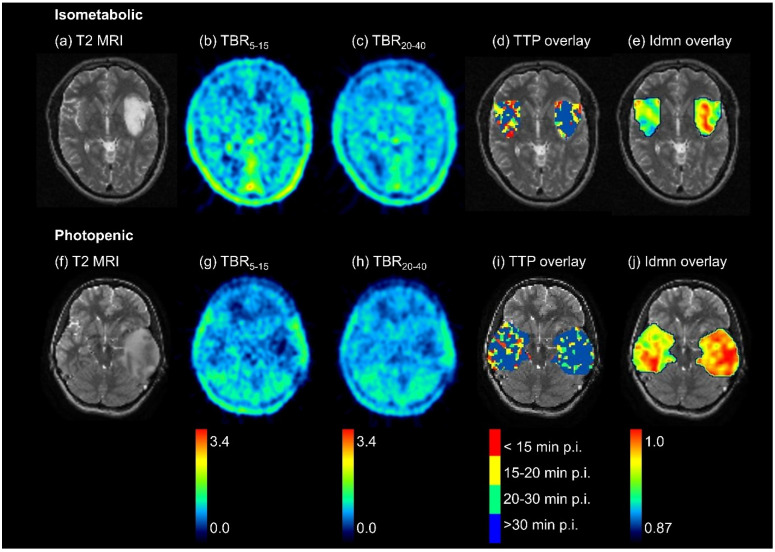
Example of an isometabolic glioma which cannot be identified visually in static TBR images (**b**,**c**) and a photopenic glioma with decreased signal in static TBR images (**g**,**h**). Tumor volumes were defined in T2-weighted MRI images (**a**,**f**). Dynamic analyses reveal differences between tumor and the mirrored volume in healthy tissue with predominantly late TTP (blue in TTP overlay, (**d**,**i**)) and higher uniformity of TTP values according to the feature inverse difference moment normalized (Idmn, (**e**,**j**)) in the tumor volume. For visualization, the 3D feature map for Idmn was calculated on a voxel-wise basis, taking into account a volume of 5 × 5 × 5 voxels around the centering voxel.

When analyzing the TTP images, the tumor VOI showed higher homogeneity and higher TTP values than the healthy tissue. A total of 14 out of 18 first-order features (78%) and 50 out of 75 texture features (67%) presented significant differences supporting these findings. This is exemplarily visualized in Figure 2 for an isometabolic glioma.

### 3.4. Differences between Photopenic Tumor and Healthy Tissue

In standard TBR_20–40_ images, in the case of photopenic gliomas, most of the significant features were also first-order parameters (10/18, 55%). As expected, the features indicated a lower magnitude of voxel intensities and lower uniformity within the photopenic gliomas compared to healthy tissue.

The early TBR_5–15_ images showed comparable findings. Significant first-order features (10/18, 56%) included, for example, simple mean and median values, which were decreased within photopenic gliomas. Texture features such as dependence non-uniformity also indicated a lower uniformity of early TBR_5–15_ images in the tumor.

In TTP images, only the following first-order features were significant (4/18, 22%): energy, total energy, 10 percentile, and variance. Overall, the tendency of radiomic features derived from TTP images suggested a higher TTP and a lower variance in the tumor.

### 3.5. Differentiation of Tumor from Healthy Tissue Using Logistic Regression

Classification results using logistic regression are provided in Table 2. Despite significant differences between tumor and non-tumor, the AUC values for the direct differentiation between both groups remained low. Better results were obtained for univariate analyses. Here, an AUC of 0.72 ± 0.14 could be reached for isometabolic gliomas using the feature high gray level run emphasis derived from TTP images and an AUC of 0.86 ± 0.15 for photopenic gliomas using the first order feature 10 percentile derived from TBR_5–15_ images. In univariate analyses, texture features derived from TTP images and a few first-order features derived from TBR images yielded the highest scores for differentiation of isometabolic tumors from healthy tissue. In the case of photopenic tumors, first-order parameters derived from TBR_5–15_ and TBR_20–40_ images yielded the highest univariate AUC values. Results from univariate analyses are provided in Supplementary Table S2.

## 4. Discussion

The characteristics of [^18^F]FET-negative gliomas are not yet clarified. This study systematically evaluates the radiomic characteristics of [^18^F]FET-negative gliomas within a group of newly diagnosed gliomas who had undergone a dynamic [^18^F]FET PET scan and were visually classified as [^18^F]FET-negative. Recent studies showed the usefulness of radiomic features derived from [^18^F]FET PET images for improved tumor classification [12,13,21], differentiation of treatment-related changes from tumor progression [9] or local relapse [22,23], or for survival prediction [24]. These studies show that radiomic analyses enable the extraction of additional clinically relevant information from images complementing simple VOI statistics and thus improve diagnostic and prognostic performance. However, radiomic analyses have not yet been applied to [^18^F]FET-negative gliomas. Hence, the aim of this study was to investigate if radiomic analyses can provide more information in [^18^F]FET-negative gliomas than is visually possible for a physician and if differences are found between tumor tissue and healthy background. For this purpose, radiomic features were extracted from standard 20–40 min p. i. TBR images, from early 5–15 min p. i. TBR images and TTP images were derived from dynamic analysis and were then compared between the T2-hyperintense tumor and its mirrored VOI in healthy background.

The largest fraction of significantly different features were obtained for features derived from TTP images. Similar results were found for isometabolic gliomas upon exclusion of photopenic gliomas, i.e., [^18^F]FET-negative gliomas, which cannot be visually identified.

Interestingly, only a small fraction of significantly different features derived from static TBR images were found for isometabolic gliomas. Surprisingly, the first-order features derived from TBR_20–40_ images quantifying the magnitude of voxel values were significantly increased in tumors compared to healthy tissue, although this could not be identified visually. Further, features quantifying heterogeneity, for example, size zone uniformity, showed a higher variability of size zone volumes implying that the uptake pattern in tumor tissue is more heterogeneous than in healthy tissue. Such heterogeneity is not directly obvious when inspecting static TBR images and could, for example, reflect the presence of small sub-volumes that exhibit either slightly increased or decreased uptake. In the case of TBR_5–15_ images, only three of all radiomic features were significantly different, reflecting an increased asymmetry of the intensity histogram in tumor tissue. Even more important for the differentiation of isometabolic tumors from healthy tissue were features derived from TTP images. A large fraction of significantly different features (64%) was observed, indicating not only higher homogeneity in tumor but also larger TTP values compared to healthy tissue. Both observations may be related to the inhibition of vasculo- and angiogenesis signaling pathways and, thus, a reduced blood volume fraction in *IDH* mutant gliomas, which represents the majority of our patient cohort, while normal vasculature is present in healthy tissue [25,26]. Since blood time-activity curves are characterized by an early peak after radiotracer bolus injection, the reduced tumor vascularization and thus lower blood volume result in a decreased number of voxels with an early peak, which corresponds to an increased number of voxels with TTP above 30 min p. i. The predominant assignment of voxels to the group with an ascending kinetic (TTP > 30 min p. i.) directly leads to a more homogeneous distribution of TTP values within the tumor. When using univariate classification, the texture features derived from TTP images reached the highest AUC scores.

For photopenic gliomas, the percentages of significantly different features were comparably low for all three image types. The radiomic data derived from standard TBR_20–40_ and early TBR_5–15_ showed, as expected, a lower magnitude of voxels. This finding can also be visually identified. Whether the overall decreased uptake can be attributed to a generally reduced vascularization and thus a low tracer availability in tumor tissue, an increased wash-out, or to some other phenomenon needs to be evaluated further. In accordance with the results of isometabolic gliomas, radiomic features derived from TBR images insinuate an increased heterogeneity of uptake patterns in photopenic tumor tissue. Consistent with the findings in isometabolic gliomas, the TTP was found to be longer in photopenic tumor tissue than in healthy tissue, and the variance was decreased. The first-order TBR features, using univariate classification, showed the highest AUC scores.

Several limitations of the study need to be addressed. Differentiation of tumor from healthy tissue has been performed in this study by defining tumor volumes within T2/FLAIR MRI images, which itself is already indicative of the presence of a tumor lesion. Furthermore, unlike the Wilcoxon test, the classification does not consider the paired nature of the tumor and the corresponding mirrored background VOI of each patient. Furthermore, the robustness of statistical analyses and machine learning might be affected by the small number of patients. Before a potential application for clinical patient assessment, data pooling also from different centers in combination with feature harmonization techniques should be used to improve model generalizability. Of further interest might be a voxel-based classification of tumor and healthy tissue using voxel-wise radiomic and parametric maps derived from dynamic [^18^F]FET PET data alone. Moreover, it might be interesting to compare [^18^F]FET-negative gliomas with non-neoplastic lesions such as Multiple Sclerosis lesions or after ischemia. For a classification of [^18^F]FET-negative lesions using tumor and non-neoplastic lesions as comparison groups, ratios of radiomic features derived from the lesion VOI and the mirrored VOI in healthy tissue might be considered. In this context, it might be interesting to compare results from dynamic PET with results from radiomic analyses performed on sophisticated MRI sequences such as diffusion-weighted imaging, dynamic susceptibility contrast, or dynamic contrast-enhanced MRI [27]. These sequences might also provide further insight into the underlying differences between [^18^F]FET-negative and [^18^F]FET-positive gliomas by quantifying, for example, blood volume or diffusion properties of the tumor. Another limitation resulting from the use of a mirrored background VOI for comparison is that the shape of the tumor and healthy tissue VOIs were identical; thus, shape features had to be excluded from analyses.

While dynamic PET imaging is time-consuming and kinetic analysis may require dedicated software solutions, it has huge potential for increasing clinical performance, which might be further enhanced by the application of total-body PET/CT devices [28]. The increased sensitivity due to the long axial field-of-view enables the acquisition of shorter time frames, i.e., improved temporal resolution, and thus, e.g., the reduction of motion artifacts and the extraction of more precise information for the diagnosis, but also for targeted therapy. Furthermore, there is conceivable clinical utility in the ability to extract high-quality information even from very late time points after radiotracer injection. Moreover, it will be interesting to evaluate the relevance of total-body PET/CT for noninvasive pharmacokinetic modeling utilizing an image-derived input function derived from the ventricle or aorta [29].

## 5. Conclusions

Several radiomic features which allow for differentiating [^18^F]FET-negative tumor tissue from healthy tissue using dynamic [^18^F]FET PET information could be identified. A decreased [^18^F]FET PET signal in tumors visually classified as photopenic gliomas could be confirmed using radiomic analyses, where first-order features from static images presented the highest significance. Even visually not recognizable differences could be observed in the time-dependent uptake pattern of isometabolic gliomas, where texture features derived from TTP images were most relevant. Yet, the underlying pathophysiological mechanisms and the clinical applicability of dynamic [^18^F]FET PET information for diagnostic purposes in [^18^F]FET-negative gliomas need to be further addressed in future studies.

## Figures and Tables

**Table 1:** Number of significant features (*p* < 0.05) according to Wilcoxon test for paired non-parametric variables. The total number of included features per image was 93 (pyradiomics default excluding shape features). Numbers are provided as absolute values and percentages.

Image	Whole Cohort (n = 46)	Isometabolic (n = 29)	Photopenic (n = 17)
TBR_20–40_	5 (5%)	11 (12%)	19 (20%)
TBR_5–15_	14 (15%)	3 (3%)	25 (27%)
TTP	64 (69%)	64 (69%)	24 (26%)

**Table 2:** Mean and standard deviation of AUC values obtained using logistic regression classification with 50-repeated 5-fold CV.

Included features	Whole Cohort	Isometabolic	Photopenic
TBR_20–40_	0.54 ± 0.14	0.55 ± 0.15	0.66 ± 0.20
TBR_5–15_	0.65 ± 0.13	0.56 ± 0.13	0.79 ± 0.17
TTP	0.61 ± 0.12	0.64 ± 0.14	0.55 ± 0.20
All	0.64 ± 0.13	0.67 ± 0.15	0.80 ± 0.20
Univariate	0.69 ± 0.12	0.72 ± 0.14	0.86 ± 0.15

## Data Availability

The data presented in this study are available on request from the corresponding author. The data are not publicly available due to ethical restrictions.

## Supplementary Materials

The following supporting information can be downloaded at: https://www.mdpi.com/2072-6694/14/19/4860/s1, Table S1: Wilcoxon test results; Table S2: Univariate AUC results.

## References

[1] la Fougère C., Suchorska B., Bartenstein P., Kreth F.W., Tonn J.C. (2011). Molecular imaging of gliomas with PET: Opportunities and limitations. Neuro-Oncol..

[2] Lohmann P., Stavrinou P., Lipke K., Bauer E.K., Ceccon G., Werner J.M., Neumaier B., Fink G.R., Shah N.J., Langen K.J. (2019). FET PET reveals considerable spatial differences in tumour burden compared to conventional MRI in newly diagnosed glioblastoma. Eur. J. Nucl. Med. Mol. Imaging.

[3] Law I., Albert N.L., Arbizu J., Boellaard R., Drzezga A., Galldiks N., la Fougere C., Langen K.J., Lopci E., Lowe V. (2019). Joint EANM/EANO/RANO practice guidelines/SNMMI procedure standards for imaging of gliomas using PET with radiolabelled amino acids and [18F]FDG: Version 1.0. Eur. J. Nucl. Med. Mol. Imaging.

[4] Floeth F.W., Pauleit D., Sabel M., Stoffels G., Reifenberger G., Riemenschneider M.J., Jansen P., Coenen H.H., Steiger H.J., Langen K.J. (2007). Prognostic value of O-(2-18F-fluoroethyl)-L-tyrosine PET and MRI in low-grade glioma. J. Nucl. Med..

[5] Unterrainer M., Schweisthal F., Suchorska B., Wenter V., Schmid-Tannwald C., Fendler W.P., Schüller U., Bartenstein P., Tonn J.C., Albert N.L. (2016). Serial 18F-FET PET Imaging of Primarily 18F-FET-Negative Glioma: Does It Make Sense?. J. Nucl. Med..

[6] Vettermann F.J., Diekmann C., Weidner L., Unterrainer M., Suchorska B., Ruf V., Dorostkar M., Wenter V., Herms J., Tonn J.C. (2021). L-type amino acid transporter (LAT) 1 expression in (18)F-FET-negative gliomas. EJNMMI Res..

[7] Habermeier A., Graf J., Sandhöfer B.F., Boissel J.P., Roesch F., Closs E.I. (2015). System L amino acid transporter LAT1 accumulates O-(2-fluoroethyl)-L-tyrosine (FET). Amino Acids.

[8] Galldiks N., Unterrainer M., Judov N., Stoffels G., Rapp M., Lohmann P., Vettermann F., Dunkl V., Suchorska B., Tonn J.C. (2019). Photopenic defects on O-(2-[18F]-fluoroethyl)-L-tyrosine PET: Clinical relevance in glioma patients. Neuro-Oncol..

[9] Lohmann P., Elahmadawy M.A., Gutsche R., Werner J.M., Bauer E.K., Ceccon G., Kocher M., Lerche C.W., Rapp M., Fink G.R. (2020). FET PET Radiomics for Differentiating Pseudoprogression from Early Tumor Progression in Glioma Patients Post-Chemoradiation. Cancers.

[10] Lohmann P., Meissner A.K., Kocher M., Bauer E.K., Werner J.M., Fink G.R., Shah N.J., Langen K.J., Galldiks N. (2020). Feature-based PET/MRI radiomics in patients with brain tumors. Neurooncol. Adv..

[11] Gillies R.J., Kinahan P.E., Hricak H. (2016). Radiomics: Images Are More than Pictures, They Are Data. Radiology.

[12] Li Z., Kaiser L., Holzgreve A., Ruf V.C., Suchorska B., Wenter V., Quach S., Herms J., Bartenstein P., Tonn J.-C. (2021). Prediction of TERTp-mutation status in IDH-wildtype high-grade gliomas using pre-treatment dynamic [18F]FET PET radiomics. Eur. J. Nucl. Med. Mol. Imaging.

[13] Lohmann P., Lerche C., Bauer E.K., Steger J., Stoffels G., Blau T., Dunkl V., Kocher M., Viswanathan S., Filss C.P. (2018). Predicting *IDH* genotype in gliomas using FET PET radiomics. Sci. Rep..

[14] Louis D.N., Perry A., Wesseling P., Brat D.J., Cree I.A., Figarella-Branger D., Hawkins C., Ng H.K., Pfister S.M., Reifenberger G. (2021). The 2021 WHO Classification of Tumors of the Central Nervous System: A summary. Neuro-Oncol..

[15] Eigenbrod S., Trabold R., Brucker D., Erös C., Egensperger R., La Fougere C., Göbel W., Rühm A., Kretzschmar H.A., Tonn J.C. (2014). Molecular stereotactic biopsy technique improves diagnostic accuracy and enables personalized treatment strategies in glioma patients. Acta Neurochir..

[16] Ludwig K., Kornblum H.I. (2017). Molecular markers in glioma. J. Neurooncol..

[17] Jansen N.L., Graute V., Armbruster L., Suchorska B., Lutz J., Eigenbrod S., Cumming P., Bartenstein P., Tonn J.C., Kreth F.W. (2012). MRI-suspected low-grade glioma: Is there a need to perform dynamic FET PET?. Eur. J. Nucl. Med. Mol. Imaging.

[18] Vomacka L., Unterrainer M., Holzgreve A., Mille E., Gosewisch A., Brosch J., Ziegler S., Suchorska B., Kreth F.W., Tonn J.C. (2018). Voxel-wise analysis of dynamic 18F-FET PET: A novel approach for non-invasive glioma characterisation. EJNMMI Res..

[19] Unterrainer M., Vettermann F., Brendel M., Holzgreve A., Lifschitz M., Zähringer M., Suchorska B., Wenter V., Illigens B.M., Bartenstein P. (2017). Towards standardization of (18)F-FET PET imaging: Do we need a consistent method of background activity assessment?. EJNMMI Res..

[20] Zwanenburg A., Leger S., Vallières M., Löck S. (2016). Image biomarker standardisation initiative. arXiv.

[21] Haubold J., Demircioglu A., Gratz M., Glas M., Wrede K., Sure U., Antoch G., Keyvani K., Nittka M., Kannengiesser S. (2020). Non-invasive tumor decoding and phenotyping of cerebral gliomas utilizing multiparametric 18F-FET PET-MRI and MR Fingerprinting. Eur. J. Nucl. Med. Mol. Imaging.

[22] Lohmann P., Stoffels G., Ceccon G., Rapp M., Sabel M., Filss C.P., Kamp M.A., Stegmayr C., Neumaier B., Shah N.J. (2017). Radiation injury vs. recurrent brain metastasis: Combining textural feature radiomics analysis and standard parameters may increase (18)F-FET PET accuracy without dynamic scans. Eur. Radiol..

[23] Lohmann P., Kocher M., Ceccon G., Bauer E.K., Stoffels G., Viswanathan S., Ruge M.I., Neumaier B., Shah N.J., Fink G.R. (2018). Combined FET PET/MRI radiomics differentiates radiation injury from recurrent brain metastasis. Neuroimage Clin..

[24] Pyka T., Gempt J., Hiob D., Ringel F., Schlegel J., Bette S., Wester H.J., Meyer B., Forster S. (2016). Textural analysis of pre-therapeutic [18F]-FET-PET and its correlation with tumor grade and patient survival in high-grade gliomas. Eur. J. Nucl. Med. Mol. Imaging.

[25] Kickingereder P., Sahm F., Radbruch A., Wick W., Heiland S., Deimling A., Bendszus M., Wiestler B. (2015). *IDH* mutation status is associated with a distinct hypoxia/angiogenesis transcriptome signature which is non-invasively predictable with rCBV imaging in human glioma. Sci. Rep..

[26] Keil V.C., Gielen G.H., Pintea B., Baumgarten P., Datsi A., Hittatiya K., Simon M., Hattingen E. (2021). DCE-MRI in Glioma, Infiltration Zone and Healthy Brain to Assess Angiogenesis: A Biopsy Study. Clin. Neuroradiol..

[27] Minh Thong P., Minh Duc N. (2020). The Role of Apparent Diffusion Coefficient in the Differentiation between Cerebellar Medulloblastoma and Brainstem Glioma. Neurol. Int..

[28] Dimitrakopoulou-Strauss A., Pan L., Sachpekidis C. (2022). Parametric Imaging With Dynamic PET for Oncological Applications: Protocols, Interpretation, Current Applications and Limitations for Clinical Use. Semin. Nucl. Med..

[29] Wang Y., Li E., Cherry S.R., Wang G. (2021). Total-body PET kinetic modeling and potential opportunities using deep learning. PET Clin..

